# Alloying and Hardness of Eutectics with Nb_ss_ and Nb_5_Si_3_ in Nb-silicide Based Alloys

**DOI:** 10.3390/ma11040592

**Published:** 2018-04-11

**Authors:** Panos Tsakiropoulos

**Affiliations:** Department of Materials Science and Engineering, University of Sheffield, Sheffield S1 3JD, UK; p.tsakiropoulos@sheffield.ac.uk

**Keywords:** eutectic, solid solution, silicide, intermetallic

## Abstract

In Nb-silicide based alloys, eutectics can form that contain the Nb_ss_ and Nb_5_Si_3_ phases. The Nb_5_Si_3_ can be rich or poor in Ti, the Nb can be substituted with other transition and refractory metals, and the Si can be substituted with simple metal and metalloid elements. For the production of directionally solidified in situ composites of multi-element Nb-silicide based alloys, data about eutectics with Nb_ss_ and Nb_5_Si_3_ is essential. In this paper, the alloying behaviour of eutectics observed in Nb-silicide based alloys was studied using the parameters ΔH_mix_, ΔS_mix_, VEC (valence electron concentration), δ (related to atomic size), Δχ (related to electronegativity), and Ω (= T_m_ ΔS_mix_/|ΔH_mix_|). The values of these parameters were in the ranges −41.9 < ΔH_mix_ <−25.5 kJ/mol, 4.7 < ΔS_mix_ < 15 J/molK, 4.33 < VEC < 4.89, 6.23 < δ < 9.44, 0.38 < Ω < 1.35, and 0.118 < Δχ < 0.248, with a gap in Δχ values between 0.164 and 0.181. Correlations between ΔS_mix_, Ω, ΔS_mix_, and VEC were found for all of the eutectics. The correlation between ΔH_mix_ and δ for the eutectics was the same as that of the Nb_ss_, with more negative ΔH_mix_ for the former. The δ versus Δχ map separated the Ti-rich eutectics from the Ti-poor eutectics, with a gap in Δχ values between 0.164 and 0.181, which is within the Δχ gap of the Nb_ss_. Eutectics were separated according to alloying additions in the Δχ versus VEC, Δχ versus <Si>, δ versus <Si>, and VEC versus <Si> maps, where <Si> = Al + Ge + Si + Sn. Convergence of data in maps occurred at δ ≈ 9.25, VEC ≈ 4.35, Δχ in the range ≈ 0.155 to 0.162, and <Si> in the range ≈ 21.6 at.% to ≈ 24.3 at.%. The convergence of data also indicated that the minimum concentration of Ti and maximum concentrations of Al and Si in the eutectic were about 8.7 at.% Ti, 6.3 at.% Al, and 21.6 at.% Si, respectively, and that the minimum concentration of Si in the eutectic was in the range 8 < Si < 10 at.%.

## 1. Introduction

Nb-silicide based alloys (also known as Nb-silicide in situ composites) are multi-element high temperature alloys. They can offer a balance of properties, and meet property goals to enable future aero engines to comply with new environmental and performance targets [1]. Alloying additions that have been reported in Nb-silicide based alloys include Al, B, Cr, Fe, Ga, Ge, Hf, Ho, Mo, Si, Sn, Ta, Ti, V, W, Y or Zr. The desirable phases in these Nb-Si based alloys are the bcc Nb solid solution (Nb_ss_) and tetragonal Nb_5_Si_3_ silicide. Other phases also can be present, such as for example C14-NbCr_2_ Laves and A15-Nb_3_X intermetallic phases.

In current aero engines, columnar grained and single crystal blades that were manufactured from Ni-based superalloys are used. Eutectic alloys with unusual highly anisotropic microstructures and properties can be manufactured. The properties of directionally solidified (DS) eutectic alloys depend on the regularity and directionality of the microstructure. DS in situ composites can be used at high stress levels at high homologous temperatures, and are capable of meeting the needs of different applications. The evaluation of DS eutectic Nb-silicide in situ composites is desirable.

In situ composites can be produced via liquid-to-solid and solid-to-solid phase transformations. In binary systems with eutectic and eutectoid reactions, in situ composites of the binary eutectics and eutectoids have been studied. The directional solidification of alloys of eutectic composition can produce in situ composites with one or more high strength phases (which are often referred to as reinforcing phases) in a metal matrix. The interface between the matrix and the reinforcing phase is formed at close to equilibrium conditions, and is stable. Microstructures can be stable at high homologous temperatures. Eutectics have been grown with ductile metal matrices and reinforcing whiskers with strengths in excess of 7 GPa [2].

Aligned composites are desirable for high-temperature applications. Those that are grown from the melt have inherent advantages compared with other types of aligned composites, because adequate bonding and low reactivity between the phases is achieved, and there are no lay-up problems or fibre matrix interactions during fabrication. 

Aligned in situ composites of near eutectic composition can be fabricated. In binary alloys of compositions well removed from the eutectic, in situ composites have been grown under high G/R ratios, where G is the temperature gradient, and R is the growth rate [3,4]. Multi-component alloys can be grown with a plane front and an aligned composite structure, provided that the interface kinetics do not pose too great a barrier to growth, the G/R ratio is sufficiently high, and the convection is sufficiently low. 

DS materials are sensitive to changes in R, G, and composition. Reactions with the mould and core(s) may be a serious problem at low R values. Elements (impurities) of low concentration C_o_ may influence (i) the stability and (ii) the properties of the DS structure. The effects of such elements may be large (i) because they can cause a macroscopically planar S/L interface to become morphologically unstable. A degeneration of an S/L interface to a non-planar configuration is more pronounced at high R values that produce finer and stronger structures, or low G values. Two ratios, which depend on the processing and alloy parameters, are important. The first is the ratio G/(RC_o_), which must be greater than some critical value to produce a controlled DS eutectic. The second is the ratio (RΔT_o_/D), where ΔT_o_ is the freezing range of the alloy, and D is the diffusivity in the melt. For G < (RΔT_o_/D), the S/L interface becomes morphologically unstable. The value of ΔT_o_ depends on composition. ΔT_o_ is small when the alloy has the eutectic composition and when the impurity concentration C_o_ is low. Off-eutectic alloys have larger ΔT_o_ values, and impurities also increase ΔT_o_. The effects of growth rate fluctuations are minimized for an alloy of eutectic composition. In off-eutectic alloy compositions, growth rate fluctuations cause changes in volume fractions of phases. If the fluctuation in growth rate is large enough, the volume fraction of one phase may go to zero. Thus, knowledge about the composition of the eutectic and element(s) with low concentration (impurities) is essential for regular DS composites. In other words, composites without irregularities in the DS structure, such as change(s) in the cross-section during DS casting, can also change R and G, and thus cause the breakdown of the regular structure. 

Research in the early 1970s on titanium matrix eutectics, where Ti was reinforced with an intermetallic phase, reported that reinforcement with 31 vol % Ti_5_Si_3_ fibres gave a considerable improvement of the Young’s modulus, compressive yield strength, and creep strength compared with existing commercial alloys [5,6].

The development of Nb-silicide based alloys has concentrated almost exclusively on cast and heat-treated alloys. Powder metallurgy (PM) alloys also have been made [7]. The non-consumable electrode arc melting of small elemental charges (0.01 kg to 0.6 kg) of pure elements in water cooled copper crucibles has been the preferred processing route for the large majority of cast alloys, owing to the limited available facilities and resources for alloy making and processing worldwide. 

In the Nb–Si binary phase diagram [8], there are eutectic and eutectoid reactions (see below in this section), which, in principle, make it possible to produce in situ Nb–Si composites via liquid-to-solid and solid-to-solid phase transformations. Research has sought to utilize these reactions for the development of Nb-silicide based alloys [9]. In the 1990s, it was demonstrated that DS in situ Nb–Si composites can be grown [1,9]. DS Nb-silicide based alloys have been produced using (i) the Bridgman method with liquid metal cooling [10,11,12,13], (ii) homemade DS facilities [14,15,16], and (iii) optical floating zone (OFZ) processing [17,18,19,20,21,22]. Typical microstructures of DS Nb-silicide based alloys produced using (i) to (iii) are shown in [10,11,12,13,14,15,17,21].

The fabrication of aligned Nb_ss_ and Nb_5_Si_3_ in situ composites of multi-element Nb-silicide based alloys is highly desirable. Aligned microstructures with Nb_ss_ and Nb_5_Si_3_ could be produced using the directional solidification of eutectics that contain these phases. For the solidification processing of such in situ eutectic composites, data about the eutectics with Nb_ss_ and Nb_5_Si_3_ that can form in Nb-silicide based alloys is required. The following discussion will highlight that such data is currently either non-available or severely limited. 

Why it is possible to fabricate in situ composites of Nb-silicide based alloys? Why is there interest in eutectics with Nb_ss_ and Nb_5_Si_3_? To answer these questions, one needs to revisit what is known about eutectic(s) and eutectoid reactions in the Nb–Si binary system. In the Nb-rich part of the equilibrium Nb–Si binary phase diagram, the eutectic and eutectoid reactions are L → Nb_ss_ + Nb_3_Si (stable eutectic), and Nb_3_Si → Nb_ss_ + αNb_5_Si_3_, respectively [8]. The latter is very sluggish. In binary Nb–Si alloys, the former reaction can be suppressed under rapid solidification conditions, and replaced by the metastable eutectic reaction L → Nb_ss_ + βNb_5_Si_3_ [23,24,25]. The phase diagram used in [23] to show the metastable extension of the Nb_5_Si_3_ liquidus to form the metastable eutectic indicated stable eutectic for a liquid composition at Si = 18.7 at.%, and a metastable eutectic at Si ≈ 20 at.%. For the stable eutectic in the binary phase diagram, the reported values of the Si concentration of the liquid are in the range 15.3 at.% to 18.7 at.% [26,27]; in other words there is disagreement about the composition of the stable eutectic. There is also disagreement about the temperature of the eutectoid reaction for which the high and low temperatures of 2043 K and 1939 K, respectively, have been reported [28,29]. The concentration of Si in the metastable eutectic estimated from the metastable extension of the Nb_5_Si_3_ liquidus depends on the Nb–Si binary phase diagram that is used. 

The tetragonal Nb_5_Si_3_ silicide is preferred for Nb-silicide based alloys owing to its superior properties compared with Nb_3_Si and hexagonal γNb_5_Si_3_. The two phase Nb + αNb_5_Si_3_ area in the Nb–Si binary has a composition range from 0.6 at.%. to 37.5 at.%. This gives flexibility to form Nb + Nb_5_Si_3_ composites that are stable above the envisaged surface temperature of the new alloys in service (T_service_ ≤ 1673 K) and with different volume fractions of the phases. 

Nb-silicide based alloys have been developed that have met the toughness or creep property goals or significantly closed the gap with the oxidation property goal. These are multi-element Nb–Si-based alloys. Some of the alloying additions provide solid solution strengthening to the Nb_ss_ (for example, Mo, Ta, Ti, and W). Meanwhile, other elements suppress pest oxidation and improve oxidation at high temperatures (for example, Al, B, Cr, Fe, Ge, Hf, Sn, and Ti); other elements suppress the stable eutectic and replace it with the metastable one (for example, Al, Mo, Sn, Ta, and W), and other elements stabilise tetragonal Nb_5_Si_3_ (for example, Al, Cr, Mo, Ta, and W) and improve creep (Mo, Ta, and W). Phase diagrams provide data about eutectic and eutectoid reactions. However, for the design and development of Nb-silicide based alloys, such data is limited. For example, there are no phase diagrams for the Nb-Si-Ta, Nb-Si-Y ternary systems [30], no or limited data about the liquidus projections of the Nb-Si-Al, Nb-Si-Hf, Nb-Si-Mo, Nb-Si-V, Nb-Si-W, Nb-Si-Zr ternary systems [30], and there are disagreements about the Nb-Ti-Si and Nb-Cr-Si liquidus projections [31,32,33,34,35,36,37]. 

Recently, the alloying of Nb_ss_ and tetragonal Nb_5_Si_3_ was reported in [38,39]. The study of the solid solution [38] used the parameters ΔH_mix_ (enthalpy of mixing), ΔS_mix_ (entropy of mixing (VEC (valence electron concentration), δ (parameter related to atomic size), Δχ (parameter related to electronegativity) and Ω = T_m_ ΔS_mix_/|ΔH_mix_|. The capital letter Q was used instead of Ω for the ratio T_m_ΔS_mix_/|ΔH_mix_|in [38] to avoid confusion with the term Ω_ij_ in the definition of ΔH_mix_. The above parameters are used in the study of the so-called high entropy alloys. References for publications on high entropy alloys are given in [38]. In [38], the alloying behaviour of the solid solution was described well by the parameters δ, Δχ, and VEC. The study of tetragonal Nb_5_Si_3_ [39] also showed that the parameters Δχ and VEC described its alloying behaviour, and that the changes of the hardness of the alloyed Nb_5_Si_3_ were related to the VEC parameter. 

What can we learn about the Si concentration of eutectics that contain Nb_ss_ and Nb_5_Si_3_ from the available data for Nb-silicide based alloys? For these eutectics, would it be possible to deduce (i) the maximum and minimum concentrations of Si in the eutectic, (ii) the total concentration of simple metal and metalloid elements in the eutectic, (iii) the minimum and maximum concentrations of other alloying additions in the eutectic (see the discussion about C_o_ and ΔT_o_ earlier in this section), (iv) the dependence of the concentration of refractory metals on the Si concentration in the eutectic, and (v) whether the hardness of the eutectics is related to the VEC parameter? 

The motivation for the research presented in this paper was to answer the above questions. The structure of the paper is as follows. First, the values of the aforementioned parameters will be given for eutectics with Nb_ss_ and Nb_5_Si_3_, and compared with data for Nb-silicide based alloys and their solid solutions. Then, the relationships between them will be discussed. The focus will then be on relationships between parameters, and the concentration of simple and metalloid elements in the eutectic. Next, the relationships between the concentrations of solute additions and the Si concentration in the eutectic will be discussed. Finally, the hardness of the eutectics will be considered.

## 2. Methodology

The available experimental data for the eutectics with Nb_ss_ and Nb_5_Si_3_ in Nb-silicide based alloys from [40,41,42,43,44,45,46,47,48,49,50,51,52,53,54,55,56] was used to seek out relationships between the parameters ΔH_mix_, ΔS_mix_, VEC, δ, Δχ, and Ω, and between these parameters and the hardness of eutectics. The actual compositions of eutectics with Nb_ss_ and Nb_5_Si_3_ were the essential requirement in order to calculate the parameters of the eutectic. The equations that were used to calculate the parameters H_mix_, S_mix_, VEC, δ, Δχ and Ω were given in [38]. The data for the properties of elements was from the same sources as in [38]. 

All of the eutectics studied in this paper were observed in the cast microstructures of Nb-silicide based alloys that had been prepared using arc melting with non-consumable tungsten electrodes in an inert atmosphere with water-cooled copper crucibles. The phases in the cast microstructures of the alloys were identified using XRD (Siemens D5000, Hiltonbrooks Ltd., Crewe, UK) and JCPDS data (International Centre for Diffraction Data), and quantitative microanalysis [40,41,42,43,44,45,46,47,48,49,50,51,52,53,54,55,56]. For the latter, electron probe microanalysis (EPMA) was used in a JEOL 8600 EPMA (JEOL Ltd., Tokyo, Japan) equipped with energy-dispersive and wavelength-dispersive spectrometers and the Oxford Link INCA software (Oxford Instruments plc, Abingdon, UK). Carefully polished standards of high purity of Nb, Si, and the other alloying element additions (Al, Cr, Fe, Ge, Hf, Mo, Sn, Ta, Ti, V, W, Y, and Zr) were used. At least 10 analyses were performed for each eutectic area in an alloy. Each specimen had been carefully polished, and was not etched. The hardness of eutectics was measured using a CV-430 AAT automatic hardness-testing machine. The load that was used was 10 kg, and was applied for 20 seconds. The indentations were made only on the eutectic areas, and covered a larger area relative to inter-lamellar spacing. At least 10 measurements were taken for each phase. The hardness measurements were taken from eutectics with similar inter-lamellar spacing in the order of micrometres. No new experimental data were created during the course of this study.

Eutectics with Nb_ss_ and Nb_5_Si_3_ have been observed in Nb-silicide based alloys with/out Ti addition, and with simple metal and metalloid element additions and other transition and refractory metal additions. The alloys belong in different alloy systems. Eutectics with Nb_ss_ and Nb_5_Si_3_ have been reported in ternary Nb-Si-X (X = Ga, Ge, Mo, Sn) [43,45,57,58]; quaternary Nb-Si-Hf-X (X = Al, Cr, Sn, W) [40,42,49], Nb-Si-Mo-X (X = Al, W) [21,42], Nb-Si-Ge-X (X = Al, Cr) [47,48], Nb-Ti-Si-X (X = Al, Ge, Sn) [46,59]; quinary Nb-Si-Hf-Sn-Al [49], Nb-Si-Hf-Mo-Ta [41], Nb-Ti-Si-Al-Cr [59], Nb-Ti-Si-Sn-X (X = Al, Cr, Fe, Hf) [44,51,52], Nb-Ti-Si-Al-X (X = Ge, Hf) [48], and Nb-Ti-Si-Mo-W [41,42] alloys; and higher order alloys, such as for example Nb-Ti-Si-Al-Cr-X (X = Ge, Hf, Sn, Ta) [10,12,13,15,19,50,53,60], Nb-Ti-Si-Hf-Mo-W [61], Nb-Ti-Si-W-Ge-Sn-X (X = Mo, Ta) [50,53], Nb-Ti-Si-Al-Cr-Mo-W-Ge-Sn [50,53], Nb-Ti-Si-Al-Cr-Ge-Y [55], Nb-Ti-Si-Al-Cr-Hf-B-Y [14,16], and Nb-Ti-Si-Al-Cr-Hf-Ta-Ho [20] alloys. Eutectics with Nb_ss_ and Nb_5_Si_3_ have been observed in boron containing Nb-silicide based alloys, but there is no data for the chemical composition of such eutectics (see discussion of Figures 6 and 13 in the next section). 

Both Nb_ss_ and Nb_5_Si_3_ in Nb-silicide based alloys can be alloyed; for example, Nb-32.4Ti-1.4Si-5.8Cr-2.5Al-2.8Fe-4.9Sn-1.3Hf and Nb-34.1Si-20.7Ti-9.6Hf-3.7Al-1.8V-0.4Cr-0.4Sn are actual average chemical compositions (at.%) respectively of a solid solution and a Nb_5_Si_3_ silicide in two different Nb-silicide based alloys. Some of the alloying additions partition preferably to one of these phases (for example, Sn partitions to the Nb_ss_ [43,44] and Ge partitions to the Nb_5_Si_3_ [45,46,47,48]) and other elements partition to both phases (for example, Hf and Ti). Depending on alloying additions, other phases also can form, for example A15-Nb_3_X (X = Al, Ge, Sn, Si) and C14-NbCr_2_ Laves. These phases also are alloyed; for example, Nb-25.4Ti-2.3Cr-0.7Fe-0.3Hf-10.9Sn-5.1Si-2.4Al [52] and Nb-6.6Ti-6.9Ta-4.4W-1.1Hf-44.2Cr-8.7Si-6Al-1Ge-0.6Sn [50] are actual average chemical compositions (at.%) respectively of an A15 and a Laves phase in two different Nb-silicide based alloys. 

The A15 phases are observed next to the Nb_ss_, and the Laves phases form in the last to solidify Cr-rich melt in between Nb_ss_ and Nb_5_Si_3_ dendrites [59,60]. Under backscatter electron (BSE) imaging conditions, the contrasts of Nb_ss_ and A15-Nb_3_X phase are similar. Ti-rich areas can form in Nb_ss_ and Nb_5_Si_3_. Nb_ss_/Nb_5_Si_3_ interfaces also can be Ti-rich. In Hf and Ti-containing alloys, the increase of the concentration of Ti in Nb_ss_ and/or Nb_5_Si_3_ is accompanied by an increase of the concentration of Hf. In alloys with Mo and W additions, the partitioning of these elements and Ti in the Nb_ss_ also creates problems with contrast, as Mo and W “do not like Ti in the Nb_ss_”, meaning that as the concentrations of Mo and W increase, that of Ti decreases. The variations in contrast that arise from the partitioning of solutes and the fine microstructures of the eutectics sometimes made it very difficult to confirm whether a binary Nb_ss_ + Nb_5_Si_3_ or a ternary eutectic between Nb_ss_ and Nb_5_Si_3_ and A15-Nb_3_X or C14-NbCr_2_ Laves had formed in alloys with three or more phases. In the great majority of the alloys studied in this paper binary, Nb_ss_ + Nb_5_Si_3_ eutectics were observed. All of the eutectics studied in this paper contained Nb_ss_ and Nb_5_Si_3_. No eutectics that contained Nb_ss_ and Nb_3_Si were studied in this paper.

The experimental data [40,41,42,43,44,45,46,47,48,49,50,51,52,53,54,55,56] about the actual average chemical compositions of the eutectics with Nb_ss_ and Nb_5_Si_3_ observed in alloys of the aforementioned systems was used to study the alloying behaviour of the eutectic. Compositions of eutectics with Nb_ss_ and Nb_5_Si_3_ are given in [40,41,42,43,44,45,46,47,48,49,50,51,52,53,54,55,56]. The eutectics had 1.9 < Al < 6.4 at.%, 1.3 < Cr < 7.7 at.%, 1.2 < Ge < 7.8 at.%, Hf < 8.6 at.%, 1.2 < Mo < 11.3 at.%, 8.1 < Si < 24.4 at.%, 1.2 < Sn < 5.4 at.%, 8.2 < Ti < 36.8 at.%, and 2 < W < 5.8 at.%. The total concentration of simple element and metalloid element additions in the eutectic is given in this paper as <Si> = Al + Ge + Si + Sn; i.e., the <Si> (at.%) includes the additions of Al, Ge, Si, and Sn, depending on whether Al, Ge, and Sn were present individually or simultaneously in the alloy.

## 3. Results and Discussion

The ranges of the values of the aforementioned parameters for the eutectics with Nb_ss_ and Nb_5_Si_3_ are given in Table 1, where they are compared with those of the Nb-silicide based alloys [62] and Nb_ss_ in all of the alloys [38]. As was the case in [62], the parameters were not used to predict whether the eutectics are HEAs (high entropy alloys) or whether the solid solution and/or intermetallic(s) will be stable. They were used to study alloying behaviour in the eutectics, discover if there are relationships between parameters and between parameters and solute concentrations in eutectics, and find out if the hardness of eutectics is related to specific properties. Compared with Nb-silicide based alloys [62], the eutectics had wider ranges of ΔH_mix_, ΔS_mix_, Δχ, and Ω values, the VEC range was essentially the same, the range of δ values was narrow, and some eutectics had δ values that were lower than those in the Nb-silicide based alloys. Compared with Nb solid solutions [38], the eutectics had more negative ΔH_mix_ values with a wider range, the ΔS_mix_ range was slightly wider, the ranges of the VEC, Δχ, and δ values were narrow and in the ranges of the values of Nb_ss_ in all of the alloys, and the Ω values were smaller and outside the Ω range of the solid solution. 

**Figure 1:**
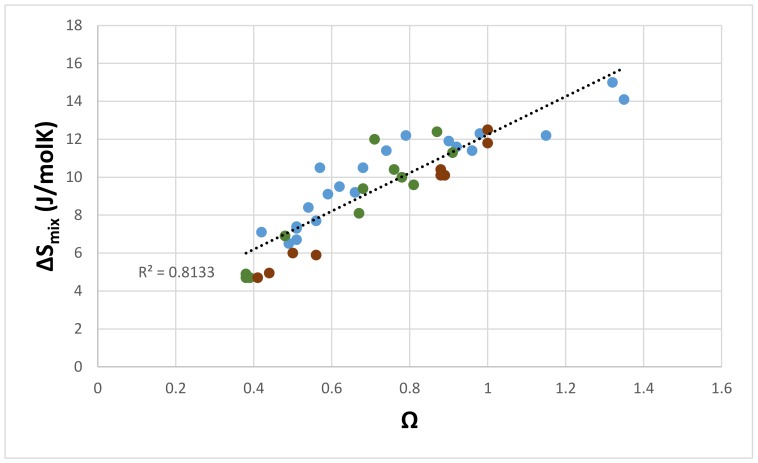
ΔS_mix_ versus Ω of eutectics with Nb_ss_ and Nb_5_Si_3_ in Nb-silicide based alloys, all data R^2^ = 0.8133, eutectics with only Sn (filled green circles) R^2^ = 0.8754, eutectics with only Ge (filled brown circles) R^2^ = 0.9863.

**Figure 2:**
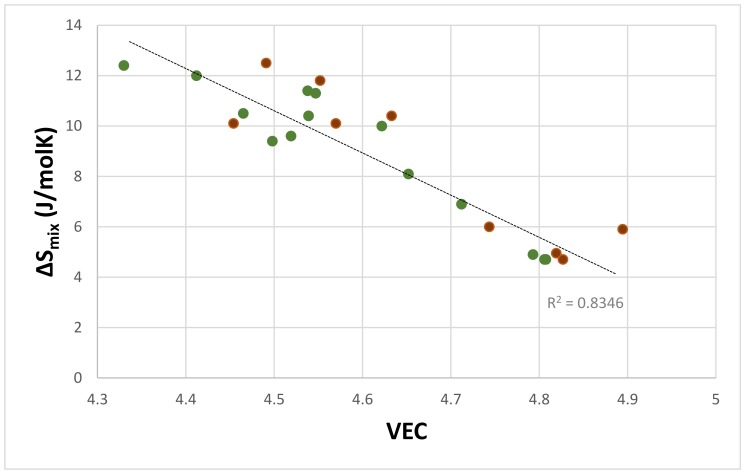
ΔS_mix_ versus VEC of eutectics with Nb_ss_ and Nb_5_Si_3_ in Nb-silicide based alloys with only Ge or Sn. All of the data R^2^ = 0.8346, eutectics with only Sn (filled green circles) R^2^ = 0.8966, eutectics with only Ge (filled brown circles) R^2^ = 0.8183.

Figure 1 and Figure 2 show the relationship between the parameters ΔS_mix_ and Ω, and ΔS_mix_ and VEC of the eutectics, respectively. Figure 1 shows that the ΔS_mix_ increases with Ω. All of the available data exhibits a linear fit with R^2^ = 0.8133, and the data subsets of the eutectics that contain only Sn (meaning <Si> = Al + Si + Sn) or only Ge (<Si> = Al + Ge + Si) exhibit better linear fits with R^2^ values of 0.8754 and 0.9863, respectively. Such a relationship was not exhibited by the ΔS_mix_ and Ω parameters of the Nb_ss_. 

Figure 2 shows that the VEC parameter increases with the decreasing ΔS_mix_ of the eutectic. The data in this figure is for eutectics in alloys with only a Ge or only an Sn addition. All of the data exhibits a linear fit with R^2^ = 0.8346. The linear fit for the eutectics with only Sn (green circles) or only Ge (brown circles), respectively, gives R^2^ = 0.8966 and R^2^ = 0.8183. Similar behaviour was not observed for the parameters VEC and ΔS_mix_ of the Nb_ss_. 

**Figure 3:**
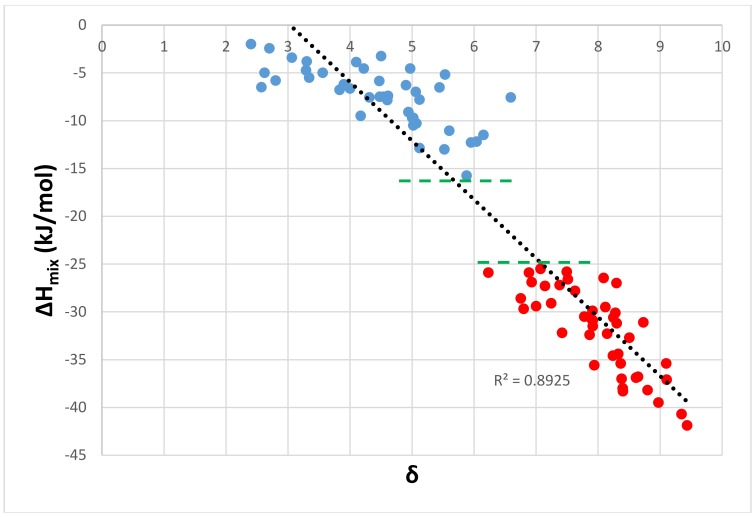
ΔH_mix_ versus δ of Nb_ss_ (filled blue circles) and of eutectics with Nb_ss_ and Nb_5_Si_3_ (filled red circles) in Nb-silicide based alloys. All of the data linear fit with R^2^ = 0.8925.

The ΔH_mix_ of the Nb_ss_ decreases (becomes more negative) as the parameter δ increases [38]. The same trend was found for the eutectics, see Figure 3. In Figure 3, the linear fit of all of the data is good (R^2^ = 0.8925), the data for the eutectics follows the trend of the data for the Nb_ss_, and the gap in the ΔH_mix_ values is shown by the horizontal dashed lines. There was also good correlation of the data in ΔH_mix_ versus Ω (R^2^ = 0.8485), and Ω versus Δχ (R^2^ = 0.8515) plots (Figures not shown) for eutectics where Ge and Sn were present simultaneously (meaning <Si> = Al + Ge + Si + Sn).

**Figure 4:**
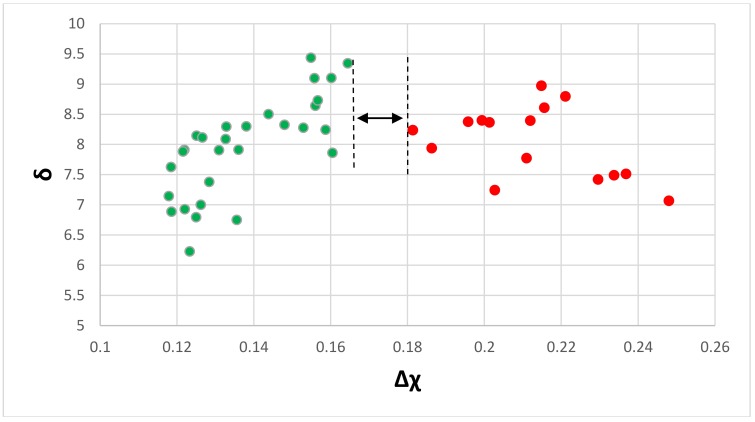
δ versus Δχ of eutectics with Nb_ss_ and Nb_5_Si_3_ in Nb-silicide based alloys. The gap in Δχ values (0.164 < Δχ < 0.181) separates eutectics with low Ti concentrations (8.3 < Ti < 13.5 at.%), which are shown with filled red circles, from those of high Ti concentration (20.5 < Ti < 45 at.%), which are shown with filled green circles.

**Figure 5:**
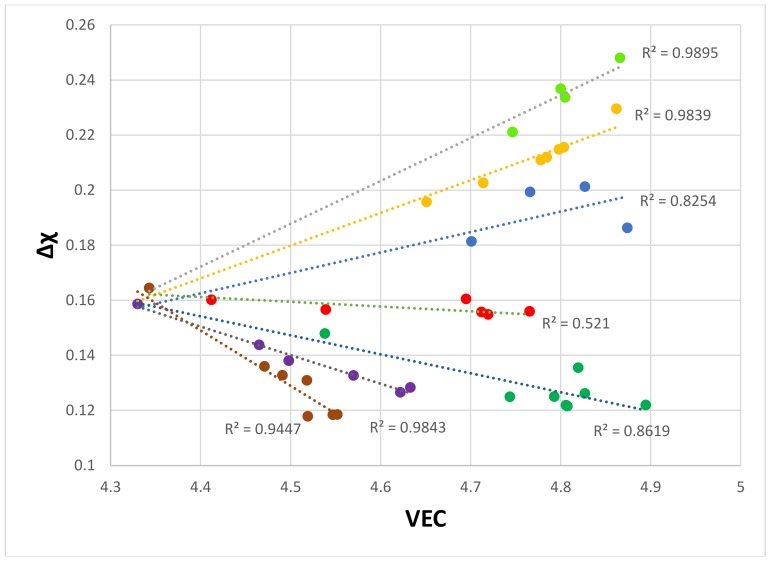
Δχ versus VEC of eutectics with Nb_ss_ and Nb_5_Si_3_ in Nb-silicide based alloys. Series a (R^2^ = 0.9447) with alloying element additions of Al, Cr, Ge, Hf, Si, Sn, Ti, and Y; series b (R^2^ = 0.9843) with alloying element additions of Al, Cr, Fe, Ge, Si, Sn, and Ti; series c (R^2^ = 0.8254) with alloying element additions of Al, Cr, Ge, Hf, Mo, Si, Sn, Ta, Ti, V, W, and Zr; series d (R^2^ = 0.9893) with alloying element additions of Al, Cr, Ge, Hf, Mo, Si, Sn, Ta, Ti, V, and W; series e (R^2^ = 0.9895) with alloying element additions of Al, Cr, Ge, Hf, Mo, Si, Sn, Ti, and W; series f (R^2^ = 0.5210) with alloying element additions of Al, Cr, Hf, Mo, Si, Sn, Ta, Ti, and V; series g (R^2^ = 0.8619) with alloying element additions of Al, Cr, Fe, Ge, Hf, Si, Sn, and Ti.

The δ versus Δχ map of the eutectics is shown in Figure 4. The partitioning of Ti in the Nb-silicide based alloys is important for the chemical composition and properties of the Nb_ss_ and Nb_5_Si_3_. For example, Ti and Cr, and Ti and Hf “like each other” in the Nb_ss_ and Nb_5_Si_3_ (meaning the concentrations of Cr and Hf increase with increasing Ti concentration in the solid solution and the silicide) but not Ti, Mo, and W in the Nb_ss_, in which as the Ti concentration increases, those of Mo and W decrease. The δ versus Δχ map separates the Ti-rich eutectics from the Ti-poor ones, and shows a gap in Δχ values between the two groups (note that in cast Nb-silicide based alloys with Ti additions, it is possible to form Ti-rich Nb_ss_ and Ti-rich Nb_5_Si_3_, [59,60]). The gap in Δχ values of the eutectics falls in the gap of Δχ values of the Nb_ss_ (Table 1). However, the δ versus Δχ map cannot separate the contributions made by different groups of alloying additions. This is possible in the Δχ versus VEC map of the eutectics, which is shown in Figure 5. The eutectics, whose data points fall in the gap of the Δχ values of the Nb_ss_, belonged in alloys where only normal Nb_ss_ was formed [38]. The alloying elements in each data series in Figure 5 are indicated in the figure caption. In the series a, as well as in the series c to f, there is no Fe; there is no Ge in series f; there is no Mo in series a, b, and g; there is no Ta in series a, b, e, and g; there is no V in series a, b, e, and g; there is no W in series a, b, f, and g; and Zr is only in series c. It should be noted that the linear fits of the data “converge” to VEC ≈ 4.35 and Δχ ≈ 0.162. The “convergence” of the data suggested that alloying elements in the eutectics in Nb-silicide based alloys might have minimum and maximum concentrations. This was confirmed by further analysis of the data for the eutectics, as shown below.

**Figure 6:**
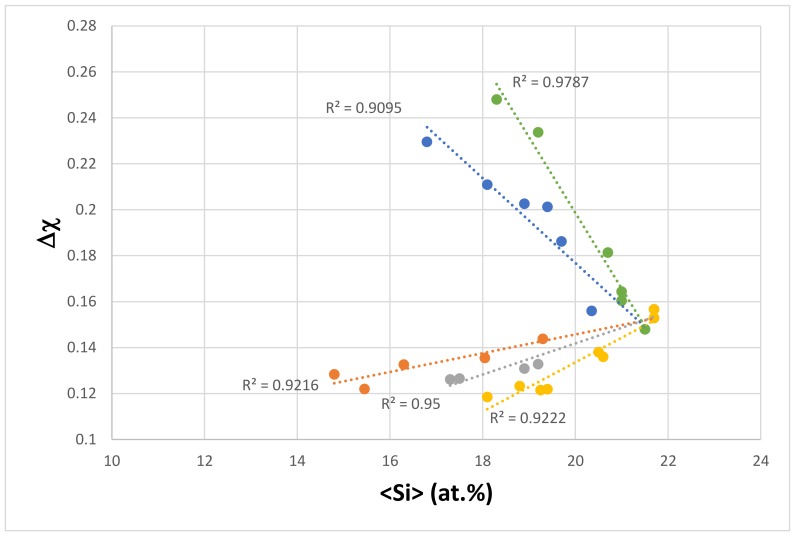
Δχ versus <Si> of eutectics with Nb_ss_ and Nb_5_Si_3_ in Nb-silicide based alloys. Series a (R^2^ = 0.9216) with alloying element additions of Al, Cr, Fe, Ge, Hf, Si, Sn, Ti, and Y; series b (R^2^ = 0.9222) with alloying element additions of Al, Cr, Ge, Hf, Si, Sn, and Ti; series c (R^2^ = 09095) with alloying element additions of Al, Cr, Fe, Ge, Hf, Mo, Si, Sn, Ta, Ti, V, W, and Zr; series d (R^2^ = 0.9787) with alloying element additions of Al, Cr, Fe, Ge, Hf, Mo, Si, Sn, Ta, Ti, and W; series e (R^2^ = 0.95) with alloying element additions of Al, Cr, Ge, Hf, Si, Sn, Ti, and Y.

**Figure 7:**
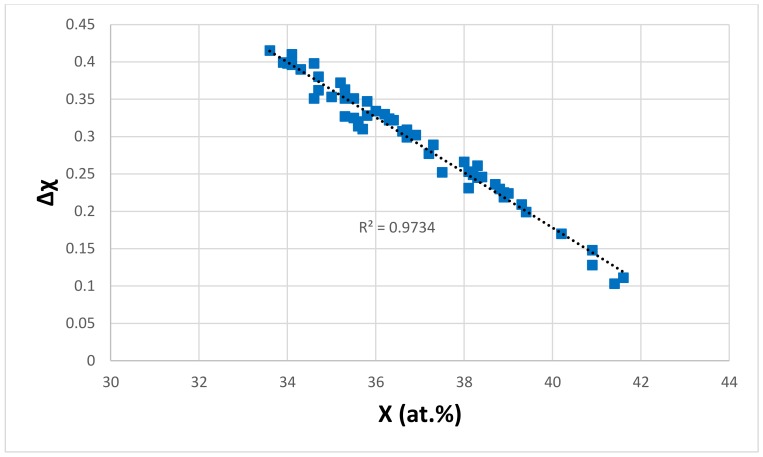
Δχ of tetragonal Nb_5_Si_3_ versus X (= Al + B + Ge + Si + Sn) in Nb_5_Si_3_ in Nb-silicide based alloys, see text.

The dependence of the parameter Δχ on the <Si> of the eutectics is shown in Figure 6. The data falls in different subsets, the alloying elements of which are given in the figure caption. All of the subsets “converge” to <Si> ≈ 21.6 at.% and Δχ ≈ 0.155. Series b and e have no Fe; there is no Mo, no Ta and no W in series a, b and e; the elements V and Zr are only in series c, and Y is only in series a and e. The trend between Δχ and <Si> for series c and d is the same as that of the parameter Δχ, and the sum of simple metal and metalloid element additions in Nb_5_Si_3_, as shown in Figure 7. Figure 7 has data for the Nb_5_Si_3_ in Nb-silicide based alloys with/out eutectics, not for Nb_5_Si_3_ in eutectics. It should be noted that (i) the data in Figure 7 includes boron-containing silicides (their data falls on the same trend as for the other simple metal and metalloid elements) and (ii) there is no data for the chemical composition of eutectics with Nb_ss_ and Nb_5_Si_3_ in boron-containing alloys (see Section 2). The data for the Δχ and <Si>_Nbss_ of the Nb_ss_, where <Si>_Nbss_ = Al + Ge + Si + Sn, showed that the Δχ of the Nb_ss_ can increase or decrease with increasing <Si>_Nbss_, depending on alloying element additions. This would suggest that in the eutectics of the series c and d in Figure 6, the composition of the eutectics was “controlled” by the silicide. Similarities between the alloying elements in the different series in Figure 5 and Figure 6 should be noted.

**Figure 8:**
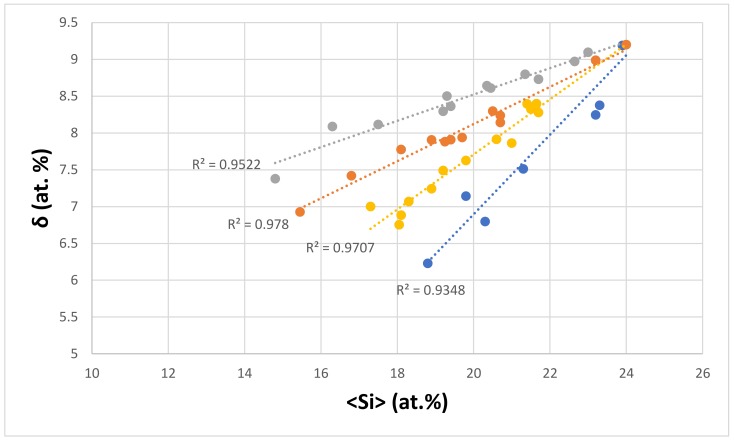
δ versus <Si> of eutectics with Nb_ss_ and Nb_5_Si_3_ in Nb-silicide based alloys. Series a (R^2^ = 0.9707) with alloying element additions of Al, Cr, Fe, Ge, Hf, Mo, Si, Sn, Ti, V, and W; series b (R^2^ = 0.9348) with alloying element additions of Al, Cr, Ge, Hf, Si, Sn, Ta, Ti, V, and W; series c (R^2^ = 0.9522) with alloying element additions of Al, Cr, Fe, Ge, Hf, Mo, Si, Sn, Ta, Ti, V, W, Y, and Zr; and series d (R^2^ = 0.978) with alloying element additions of Al, Cr, Ge, Hf, Mo, Si, Sn, Ta, Ti, and W.

The dependence of δ on the <Si> of the eutectic is shown in Figure 8. The data falls in different subsets, the alloying elements of which are given in the figure caption. Series b and d have no Fe, there is no Mo in series d, there is no Ta in series a, there is no V in series d, and Y and Zr are only present in series c. All of the series “converge” to <Si> ≈ 24.35 at.% and δ ≈ 9.25 The parameter δ increases with increasing <Si> for all of the series. Similarities between the alloying elements in the different series in Figure 5, Figure 6 and Figure 8 should be noted.

**Figure 9:**
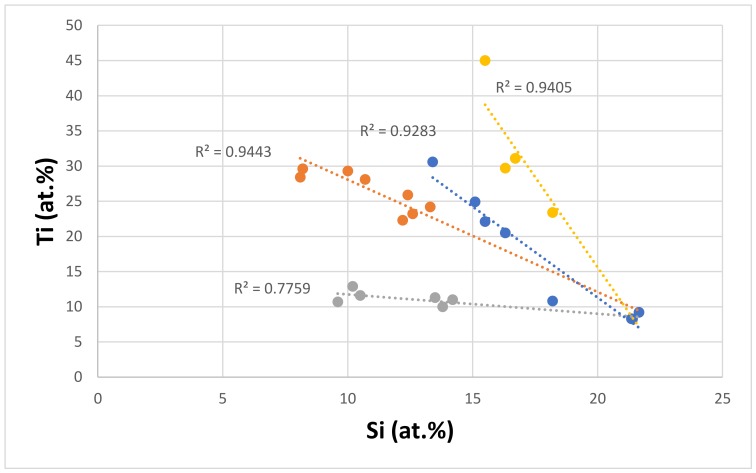
Ti versus Si of eutectics with Nb_ss_ and Nb_5_Si_3_ in Nb-silicide based alloys, series a (R^2^ = 0.9443), with alloying element additions of Al, Cr, Ge, Hf, Mo, Si, Sn, Ti, W, and Y; series b (R^2^ = 0.7759) with alloying element additions of Al, Cr, Ge, Hf, Mo, Si, Sn, Ti, V, and W; series c (R^2^ = 0.9405) with alloying element additions of Al, Cr, Fe, Hf, Mo, Si, Sn, Ti, V, and W; series d (R^2^ = 0.9283) with alloying element additions of Ge, Hf, Mo, Si, Sn, Ta, Ti, and W.

Titanium is an important addition in Nb-silicide based alloys, because it improves oxidation and toughness, and reduces density. It partitions to Nb_ss_ and Nb_5_Si_3_ [59,60] where its concentration affects that of other elements. Both phases can have Ti-rich areas that persist only in the silicide after exposure to high temperatures [59,60]. The relationship between the Ti and Si concentrations of the eutectics is shown in Figure 9. The data falls into four subsets, of which series d has no Al and Cr. All four series “converge” to Si ≈ 21.6 at.% and Ti ≈ 8.7 at.%, and show that the Si concentration in the eutectic decreases as the Ti concentration increases. The latter was also the case for the Ti and Si concentrations in Nb_5_Si_3_ in Nb-silicide based alloys with/out eutectics, but not for the Nb_ss_ where the Si concentration increases as the Ti concentration increases. 

**Figure 10:**
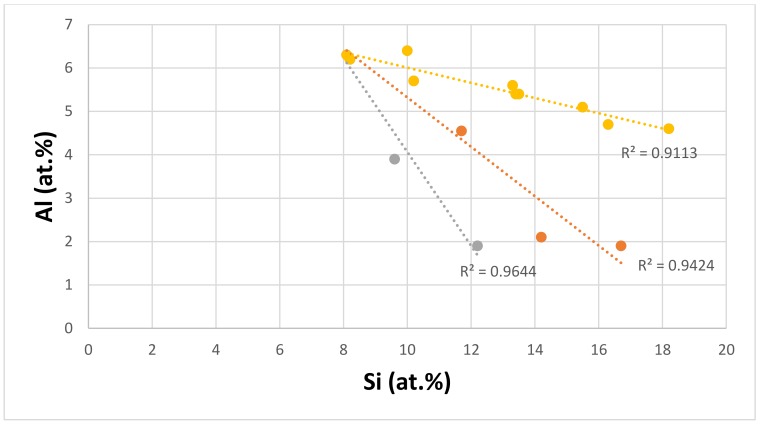
Al versus Si of eutectics with Nb_ss_ and Nb_5_Si_3_ in Nb-silicide based alloys, series a (R^2^ = 0.9644) with alloying element additions of Al, Cr, Ge, Hf, Mo, Si, Sn, Ti, W, and Y; series b (R^2^ = 0.9113) with alloying element additions of Al, Cr, Ge, Hf, Mo, Si, Sn, Ti, V, W, and Y; series c (R^2^ = 0.9424) with alloying element additions of Al, Cr, Fe, Ge, Hf, Mo, Si, Sn, Ti, and W.

Aluminium is added at low concentrations in Nb-silicide based alloys because of its adverse effect on their toughness. It reduces density and contributes to the improvement of oxidation resistance with additions of B, Ge, or Sn. It partitions to Nb_ss_ and Nb_5_Si_3_, where its concentration depends on other alloying elements [59,60]. Figure 10 shows the relationship between the Al and Si concentrations of the eutectics. The data falls in three subsets, with the alloying elements in each series indicated in the figure caption. All of the subsets of data show that as the Si concentration in the eutectic decreases, the Al content increases, which is in agreement with the data for Nb_5_Si_3_ in Nb-silicide based alloys with/out eutectics, but not with the data for the Nb_ss_. The data “converges” to Al ≈ 6.3 at.% and Si ≈ 8 at.%. Similarities between the alloying elements in the different series in Figure 9 and Figure 10 should be noted.

In Nb-silicide based alloys, Hf is added to improve the oxidation and toughness and scavenge oxygen to form hafnia. Hafnium partitions to both the Nb_ss_ and Nb_5_Si_3_, and its concentration is related to that of Ti in the two phases. The Hf concentration decreases with decreasing Si concentration in eutectics, and the data “converges” to Hf ≈ 1 at.% and Si ≈ 10 at.%. The trend of the data (figure not shown) is the same as that of the Hf and Si concentrations in the Nb_ss_ in Nb-silicide based alloys with/out eutectics. There is no correlation between the Hf and Si concentrations in Nb_5_Si_3_. 

The “convergence” of data that was shown earlier would suggest that (i) the alloying elements have maximum and minimum concentrations in the eutectics, and (ii) the maximum concentrations of Al and Si in the eutectic are about 6.3 at.% and 21.6 at.%, respectively. The minimum concentration of Ti in the eutectic is about 8.7 at.%, and the minimum concentration of Si in the eutectic is in the range of 8 at.% to 10 at.%. 

**Figure 11:**
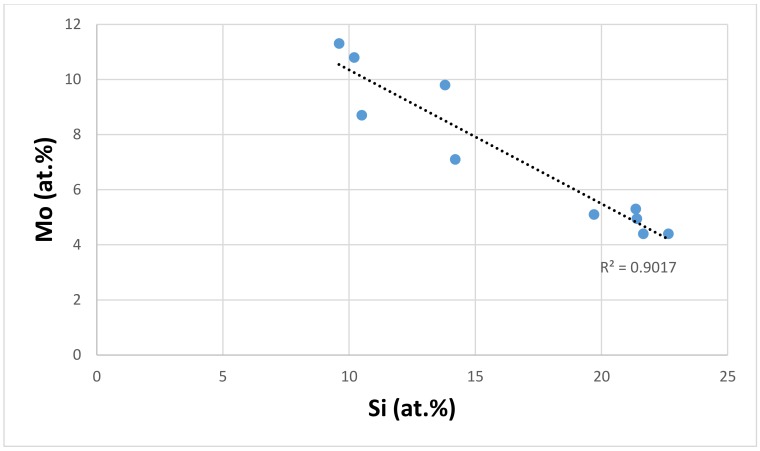
Mo versus Si of eutectics with Nb_ss_ and Nb_5_Si_3_ in Nb-silicide based alloys, elements in eutectics: Al, Cr, Ge, Hf, Mo, Nb, Si, Sn, Ti, and W.

The refractory metals Mo, Ta, and W can be present in the eutectics, and can stabilise their lamellar microstructure at high temperatures, depending on the other alloying additions in the Nb-silicide based alloy [41,42,50,53,60,61]. Their concentration in the eutectic also is related to the Si concentration of the latter. Molybdenum is chosen to demonstrate this relationship in this paper. Figure 11 shows that the Mo concentration of the eutectics decreases as the Si concentration increases. This is consistent with the data for the Nb_ss_ in Nb-silicide based alloys with/out eutectics, which shows the same trend, and also with the partitioning behaviour of Ti and Mo in the solid solution where, as the Ti concentration increases, the Mo concentration decreases. 

**Figure 12:**
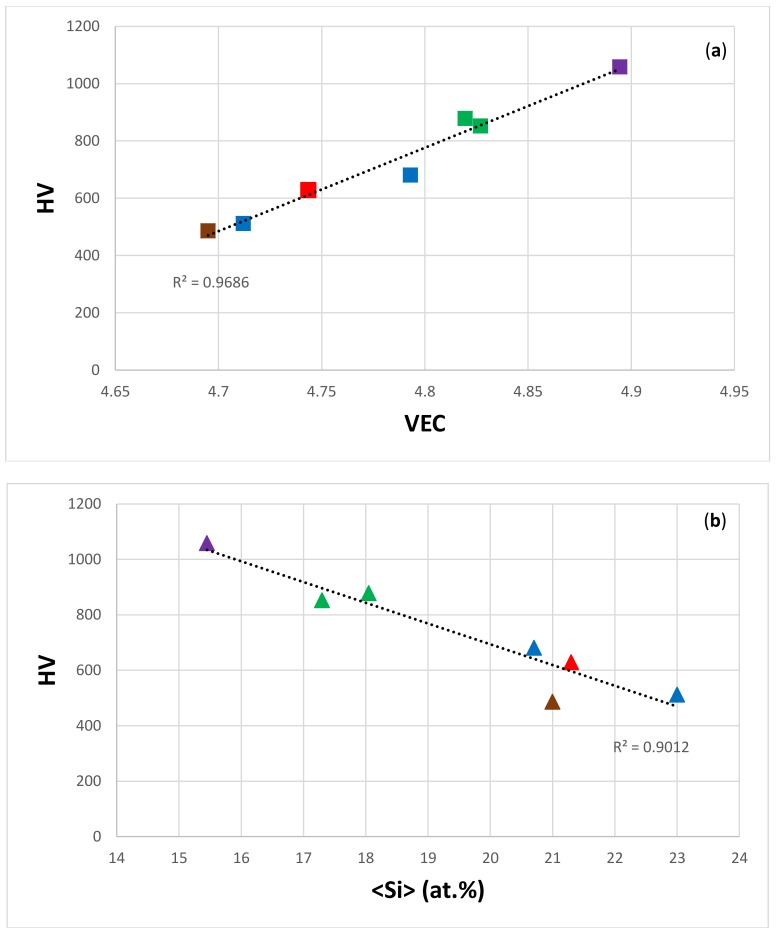
Average Vickers hardness of eutectics versus (**a**) VEC and (**b**) <Si> of eutectics. In both (**a**,**b**), data for the eutectics with Sn is shown in blue, data for the eutectics with Al is shown in brown, data for the eutectics with Al and Ge is shown in red, data for the eutectics with Ge is shown in green, and data for the eutectics with Cr and Ge is shown in purple.

**Figure 13:**
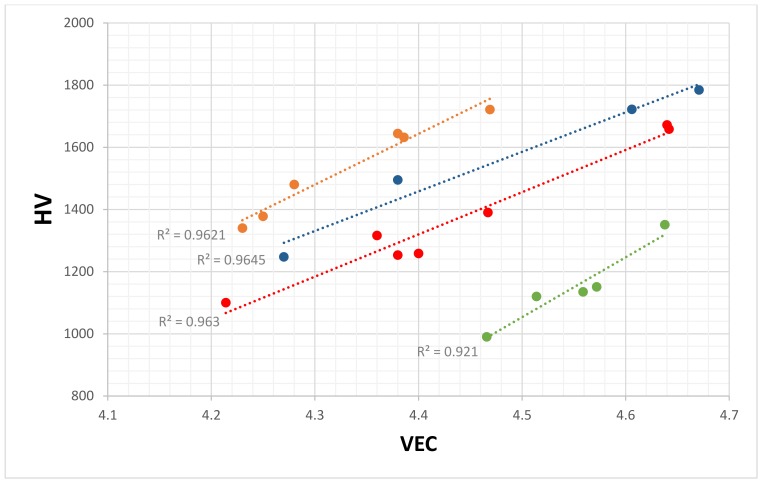
Average Vickers hardness of tetragonal Nb_5_Si_3_ silicides (not in eutectics) versus their VEC values. Alloying element additions in series a (R^2^ = 0.9621) are Al, B, Cr, Ge, Hf, Si, Sn, Ta, and Ti; the alloying element additions in series b (R^2^ = 0.9645) are Al, B, Cr, Ge, Hf, Si, and Ti; the alloying element additions in series c (R^2^ = 0.963) are Al, B, Cr, Ge, Hf, Mo, Si, Sn, Ta, Ti, and V; and the alloying element additions in series d (R^2^ = 0.921) are Al, Cr, Hf, Si, Sn, Ti, V, and W.

Data for the hardness of eutectics with Nb_ss_ and Nb_5_Si_3_ is available for the Nb-silicide based alloys without the addition of Ti, and are shown in Figure 12. The eutectics were observed in alloys of the following systems: Nb-Si-Sn, Nb-Si-Ge, Nb-Si-Hf-Al, Nb-Si-Hf-Sn, Nb-Si-Ge-Al, and Nb-Si-Cr-Ge [40,43,45,47,48,49]. The eutectics in the alloys in Figure 12 had <Si> = Si + Sn, <Si> = Si + Al, <Si> = Si + Ge, or <Si> = Si + Al + Ge. The microstructures of the Sn-containing alloys consisted of three phases, namely Nb_ss_, Nb_5_Si_3_, and A15-Nb_3_Sn, and the microstructures of the alloys without Sn addition contained only Nb_ss_ and Nb_5_Si_3_. The data points for the alloys with A15-Nb_3_Sn in their microstructures are indicated in the blue colour in Figure 12. Figure 12a shows that the hardness of the eutectics increased as the VEC increased. The same trend between hardness and VEC was observed for the hardness of the A15-Nb_3_X phases in the Nb-silicide based alloys [63], for β(Nb,Ti)_5_Si_3_ (see below), and for the hardness of tetragonal Nb_5_Si_3_, as shown in Figure 13. Note that the data in the latter figure is for the Nb_5_Si_3_ in Nb-silicide based alloys, not for the Nb_5_Si_3_ lamellae in eutectics with Nb_ss_ and Nb_5_Si_3_ in such alloys. The elements in each data series in Figure 13 are given in the caption. Mo is only in series c; W is only in series d, which does not also have B, Ge, and Ta; V is not in series a and b; and there is no Sn in series b. Eutectics with Nb_ss_ and Nb_5_Si_3_ have been observed in boron-containing Nb-silicide based alloys. There is data for the chemical composition and hardness of the 5-3 silicide in the microstructure of the latter, but not for the eutectics (see Section 2).

The data for the alloys with A15-Nb_3_Sn in Figure 12 falls on the same line as the data for the eutectics with only Nb_ss_ and Nb_5_Si_3_, and all of the data exhibits a very good linear fit (R^2^ = 0.9686). Figure 12b shows that the hardness of the eutectics decreased as their <Si> increased. The linear fit of the hardness versus the <Si> data is also good (R^2^ = 0.9012), but this was not the case when hardness was plotted against the concentration of Si in the eutectics, which varied from 10.3 at.% to 20 at.% for the alloys in Figure 12. 

**Figure 14:**
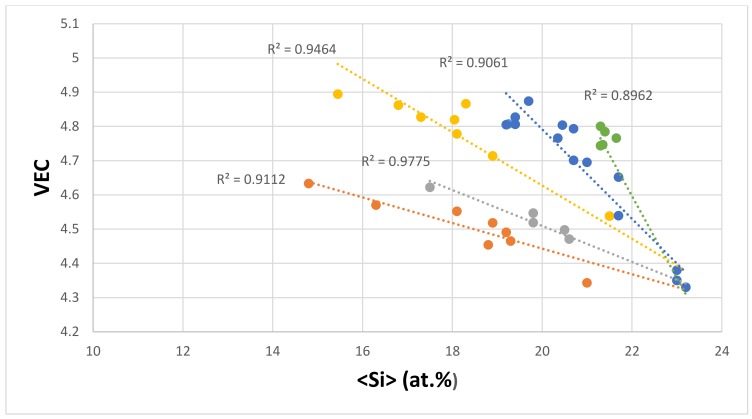
VEC versus <Si> map for eutectics with Nb_ss_ and Nb_5_Si_3_ in Nb-silicide based alloys, series a (R^2^ = 0.9464) with alloying element additions of Al, Cr, Fe, Ge, Hf, Mo, Si, Sn, Ti, V, and W; series b (R^2^ = 0.9112) with alloying element additions of Al, Cr, Fe, Ge, Hf, Si, Sn, Ti, and Y; series c (R^2^ = 0.9061) with alloying element additions of Al, Ge, Hf, Mo, Si, Sn, Ta, Ti, V, W, and Zr; series d (R^2^ = 0.8962) with alloying element additions of Al, Cr, Ge, Hf, Mo, Si, Sn, Ti, and W; series e (R^2^ = 0.9775) with alloying element additions of Al, Cr, Hf, Si, Sn, and Ti.

The ranking of the eutectics in Figure 12 in terms of their hardness from low to high values does not follow the ranking of the hardness of alloyed Nb_5_Si_3_ that was discussed in [39]. Figure 12 suggests that the VEC of the eutectic should decrease as its <Si> content increases. Indeed, this is the case as shown in Figure 14, which also confirms that the data converges to VEC ≈ 4.35 and <Si> ≈ 23 at.%. In Figure 14, there is no Cr in series c, there is no Fe in series c to e, there is no Ge in series e, there is no Mo in series b and e, and there is no Y in series a and c to e. Meanwhile, Ta is present only in series c; V is present only in series a and c; W is present only in series a, c, and d; and Zr is present only in series c. Note the similarities with the data series in Figure 14 and in Figure 6 and Figure 8. The data in Figure 6, Figure 8 and Figure 14 shows that <Si> and Δχ “converge” respectively in the ranges 21.6 at.% to 24.3 at.%, and 0.155 to 0.162. 

The hardness of intermetallics has been reported to depend on the scale of their microstructure, and to follow a Hall–Petch relationship [64,65]. Intermetallics participate in eutectics; examples include the Al/Al_2_Cu, Al/Al_3_Ni, Nb/Nb_3_Si, and Nb/Nb_5_Si_3_ eutectics. In a eutectic, the inter-lamellar spacing, the properties of the participating phases, and the interfaces between the lamellae are expected to define their mechanical properties. Refinement of eutectic microstructure can be affected by solidification conditions and/or alloying additions. When the eutectic spacing is refined, the role of interfaces becomes important. Lamellar interfaces are expected to play a major role in the deformation of eutectics. 

The improvement of properties of directionally solidified (DS) eutectics with microstructure refinement has been related to a Hall–Petch relationship for eutectics. For Al–Si eutectics, there are different hardness versus inter-lamellar spacing relationships for different silicon morphologies [66]. Mason et al. showed that the hardness of a Mo_5_Si_3_/MoSi_2_ eutectic depended on lamellar spacing in accordance with a Hall–Petch relationship, where the hardness was dependent upon the scale of the lamellae of MoSi_2_ [67]. Dislocation pile-ups are necessary for Hall–Petch strengthening. Grain boundaries impede dislocation propagation from one grain to the next. As the dislocations pile up against a grain boundary, the stress field assists dislocations to traverse the grain boundary, and thus, deformation spreads from grain to grain. Dislocations are generated during indentation for the measurement of hardness [68]. For the Mo_5_Si_3_/MoSi_2_ eutectic, Mason et al. suggested that the Mo_5_Si_3_ silicide behaved as an impenetrable barrier [67]. 

What is known about the deformation of lamellar microstructures consisting of alloyed Nb_ss_ and Nb_5_Si_3_? How would a strong or weak Nb_ss_/Nb_5_Si_3_ interface behave under mechanical loading? How important is an orientation relationship between Nb_ss_ and Nb_5_Si_3_ for the deformation of a lamellar Nb_ss_/Nb_5_Si_3_ structure? Does the morphology of Nb_5_Si_3_ depend on alloying additions? To answer these questions, we need to consider first microstructures based on the Nb–Si binary phase diagram, and then Nb–Si-based (i.e., alloyed) microstructures. Research on the deformation of Nb in Nb/Nb_5_Si_3_ micro-laminate Nb–Si binary foils [69] has highlighted the importance of layer thickness and confirmed that the deformation of Nb was dependent on the thickness of its layers in the foils. As the thickness of the latter increased, their fracture changed from ductile to brittle. This change in fracture mode was attributed to the constraint of Nb and/or changes of crack propagation rate [69]. The hardness of the Nb layers in the micro-laminate foils was 5.4 GPa, which is very close to the hardness of the 1-μm thick Nb thin films that were reported in [70], and significantly higher than the hardness of about 1.6 GPa of about 13-μm Nb particles. The high hardness of the Nb layers was attributed to “their small grain size and the narrow spaced Nb_5_Si_3_ layers, both of which acted to restrain dislocation motion in the Nb layers” [69]. Gavens et al. also reported that the estimated high average fracture strength of Nb_5_Si_3_ was typical of that of high strength ceramic fibres [69].

A study of a Nb_(001)_/αNb_5_Si_3(001)_ interface using a first-principles calculation showed that some of the Nb atoms at the interface become a part of Nb_5_Si_3_, and that the Nb–Si bonds at the interface are the likely sites for micro-cracking [71]. This study reported that the work of adhesion and fracture energy of the Nb_(001)_/αNb_5_Si_3(001)_ interface were 4.4 J/m^2^ and 33.7 J/m^2^, respectively. 

In Nb-silicide based alloys, both the Nb_ss_ and the Nb_5_Si_3_ are alloyed, and the Nb_ss_/Nb_5_Si_3_ interface can be Ti-rich (see Section 2). Also, alloying can affect the morphology of Nb_5_Si_3_, whose cross-sections can change from circular to polygonal as the entropy of fusion of Nb_5_Si_3_ increases, owing to alloying additions. The mechanical properties of [Nb_ss_]_(002)_ /[αNb_5_Si_3_]_(002)_ interfaces in a directionally solidified Nb-silicide based alloy of nominal composition Nb-24Ti-15Si-4Cr-2Al-2Hf (at.%) were studied experimentally, and with finite element modelling by Guan et al. [72]. The lower work of the adhesion and fracture energy values compared with the work of Shang et al. [71] were attributed to the alloying of the [Nb_ss_]_(002)_/[αNb_5_Si_3_]_(002)_ interface, as the latter would be expected to be rich in Ti and Hf on the silicide side and Ti, Hf, Al and Cr on the Nb side, and the different orientation relationship of the studied interface.

The data in Figure 12 is not for the same eutectic of one specific alloy composition, but for different eutectics with Nb_ss_ and Nb_5_Si_3_ in different alloys. In the latter, the phases that participated in the eutectics had different chemical compositions and different hardness, and the eutectics had similar inter-lamellar spacing, but not the same volume fractions of phases and different Nb_ss_/Nb_5_Si_3_ interface chemistries. Yet, the hardness versus the VEC data for the eutectics of these different alloys followed a remarkable linear relationship with a very good R^2^ value (=0.9686). This strong relationship is attributed to the covalent bonded intermetallic phase(s) in the eutectics, which are suggested to be the key phases that determine the hardness of the eutectics. 

In the solid solution, the bonding is delocalized, and the hardness depends on grain size, grain boundaries, and contamination by interstitials (impurities). In covalent compounds, the mobilities of dislocations are low because of the localized bonding [73]. The dependence of the hardness of covalent bonded hard materials on their shear modulus is stronger than the relationship between the hardness and their bulk modulus [74]. The latter measures the resistance to volume change, which is not the case with the hardness test, and the former is a measure of the rigidity against shape change in the hardness test. Among the various shear stiffnesses, only C_44_ represents a shape change without volume change. Thus, C_44_ provides direct information about the electronic response to shear strain [75]. Jhi et al. [75] showed that there exists a relationship between C_44_ and VEC, and hardness and VEC for different transition metal carbonitrides. For each carbonitride, the trend in the aforementioned relationships was the same, the C_44_ and hardness increased with decreasing VEC to a maximum value for VEC of about 8.4. Wang and Zhou [75] showed that the C_44_ of M_2_AlC increased with increasing VEC in the order M = Ti, Nb, V, and Cr. The polynomial fit of the data indicated a maximum value of C_44_ for a VEC of about 8.5. It was also suggested that the hardness of M_2_AlC compounds could be predicted from the correlation between C_44_ and VEC [76].

**Figure 15:**
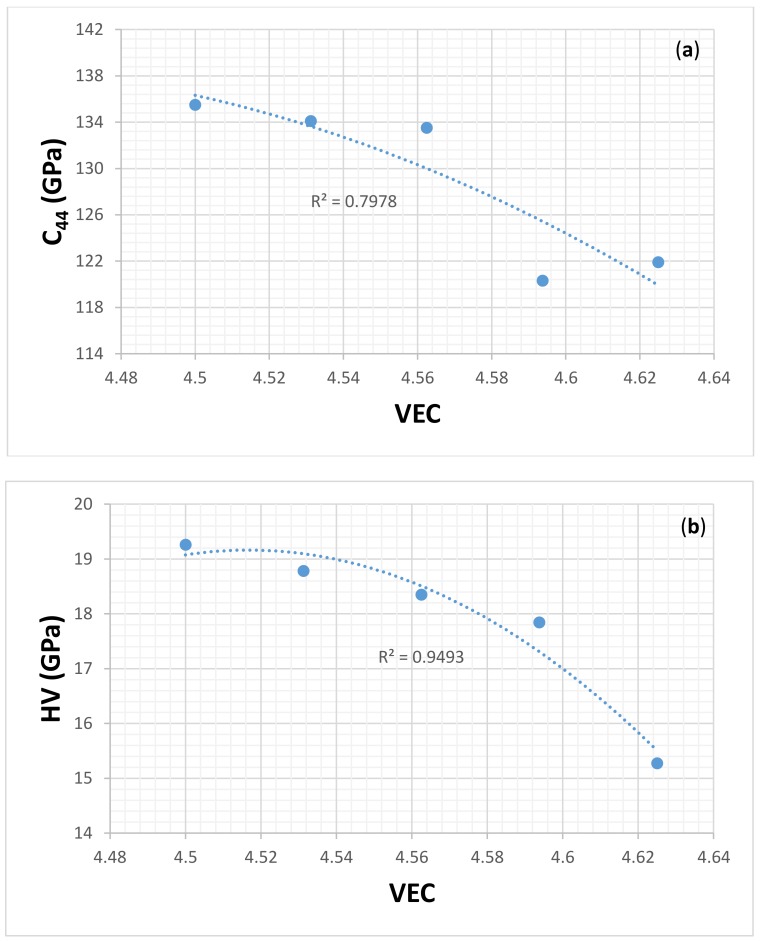
(**a**) C_44_ versus VEC and (**b**) calculated hardness versus VEC of α(Nb,Ti)_5_Si_3._

**Figure 16:**
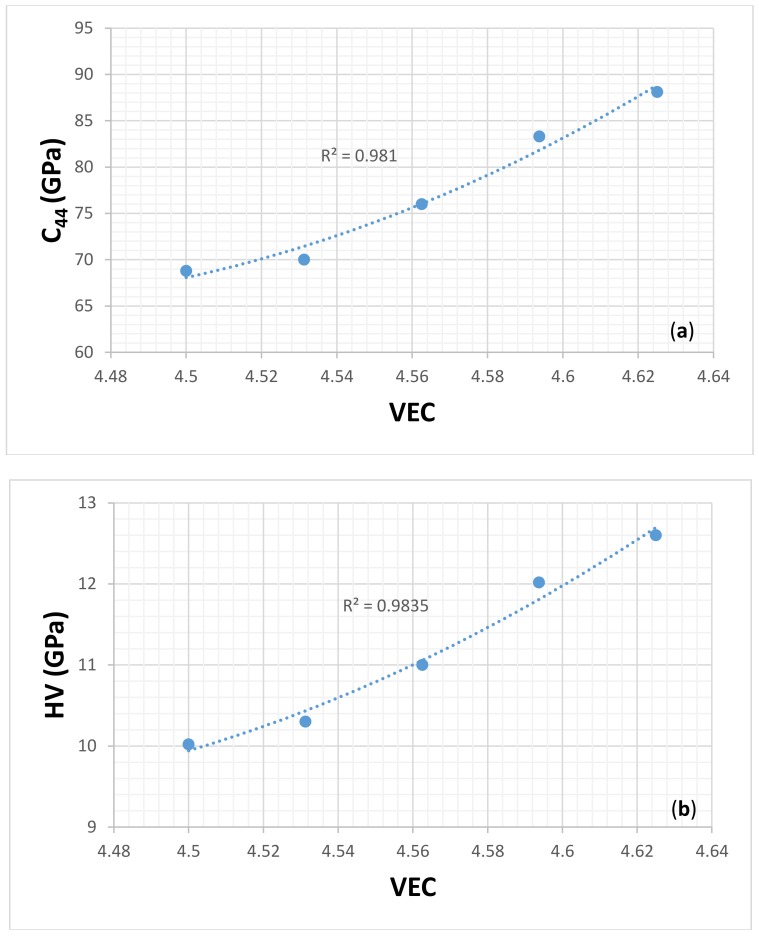
(**a**) C_44_ versus VEC and (**b**) calculated hardness versus VEC of β(Nb,Ti)_5_Si_3._

Figure 15 and Figure 16 respectively show the C44 and VEC, and hardness and VEC relationships for α(Nb,Ti)_5_Si_3_ and β(Nb,Ti)_5_Si_3_ for Ti = 0, 3.125, 6.25, 9.375, and 12.5 at.% using data for C_44_ from [77]. Unfortunately, there is no experimental data for the hardness of (Nb,Ti)_5_Si_3_ silicides. In [39], it was shown that the hardness values that were calculated using the equation HV = 2[(G/B)^2^G]^0.585^ – 3 were in better agreement with the available experimental data. The calculations of the hardness values in Figure 15 and Figure 16 used the above equation with data for shear G and bulk B moduli from [77]. Figure 15b shows that the hardness of α(Nb,Ti)_5_Si_3_ increases as the Ti concentration increases and the VEC decreases. Figure 16b shows that the hardness of β(Nb,Ti)_5_Si_3_ decreases as the Ti concentration decreases and the VEC increases. The polynomial fit of the data in Figure 15 and Figure 16a showed respectively for α(Nb,Ti)_5_Si_3_ and β(Nb,Ti)_5_Si_3_ maximum and minimum values of C_44_ for essentially the same VEC (4.426 and 4.429, respectively). Figure 15 shows that the trends between the C_44_ and VEC and hardness and VEC of α(Nb,Ti)_5_Si_3_ are the same as those reported for transition metal carbonitrides [75]. The trend between the C_44_ and VEC of β(Nb,Ti)_5_Si_3_ is the same as that reported for M_2_AlC compounds [76]. It should be noted that the trend between hardness and VEC is the same in Figure 12a and Figure 16b; the hardness increases as the VEC increases. Figure 16b is for β(Nb,Ti)_5_Si_3_. The βNb_5_Si_3_ is the 5-3 silicide in the metastable eutectic (see Introduction).

The eutectics in Figure 12 had 5-3 silicides where only Si was substituted by other simple metal and metalloid elements. There is no C_44_ data for these alloyed silicides. It is suggested that their C_44_ also correlates with VEC. It would be interesting to compare their C_44_ versus VEC and hardness versus the VEC correlations with those of (Nb,Ti)_5_Si_3_ silicides.

## 4. Conclusions

In Nb-silicide based alloys, eutectics that contain Nb_ss_ and Nb_5_Si_3_ can form. It was shown that the alloying behaviour of the eutectics, the great majority of which are binary Nb_ss_ + Nb_5_Si_3_ eutectics, can be described using the parameters ΔH_mix_, ΔS_mix_, VEC, δ, Δχ, and Ω. 

The values of these parameters were in the ranges −41.9 < ΔH_mix_ < −25.5 kJ/mol, 4.7 < ΔS_mix_ < 15 J/molK, 4.33 < VEC < 4.89, 6.23 < δ < 9.44, 0.38 < Ω < 1.35, and 0.118 < Δχ < 0.248, with a gap in the Δχ values between 0.164 and 0.181. 

Compared with Nb-silicide based alloys, the eutectics had wider ranges of ΔH_mix_, ΔS_mix_, Δχ, and Ω values, the VEC range was essentially the same, the range of δ values was narrow, and some eutectics had δ values lower than the Nb-silicide based alloys. 

Compared with Nb solid solutions, the eutectics had more negative ΔH_mix_ values within a wider range; the range of ΔS_mix_ values was slightly wider; the ranges of the VEC, Δχ, and δ values were narrow; and in the ranges of the values of Nb_ss_ in the Nb-silicide based alloys, the Ω values were smaller, and outside the range of the Ω values of the solid solution. 

There were correlations between ΔS_mix_, Ω, ΔS_mix_, and VEC for all of the eutectics. The correlation between ΔH_mix_ and δ was the same as that of the Nb_ss_. 

Specific maps of the studied parameters could describe the alloying of the eutectics. The δ versus Δχ map separated the Ti-rich from the Ti-poor eutectics, with a gap in Δχ values between 0.164 and 0.181, which is within the Δχ gap of the Nb_ss_. Eutectics were also separated according to alloying element additions in the Δχ versus VEC, Δχ versus <Si>, δ versus <Si>, and VEC versus <Si> maps where <Si> = Al + Ge + Si + Sn. 

The convergence of data in maps indicated that (i) the eutectics had δ ≈ 9.25 and VEC ≈ 4.35 and Δχ in the range ≈ 0.155 to 0.162, and <Si> in the range ≈ 21.6 at.% to ≈ 24.3 at.%, (ii) the minimum concentration of Ti, and maximum concentrations of Al and Si in the eutectic were about 8.7 at.% Ti, and 6.3 at.% Al and 21.6 at.% Si, respectively and (iii) the minimum concentration of Si in the eutectic was in the range 8 < Si < 10 at.%. 

## Figures and Tables

**Table 1:** Values of the parameters ΔH_mix_, ΔS_mix_, valence electron concentration (VEC), δ, Δχ, and Ω for all of the Nb-silicide based alloys and their solid solutions, and for the eutectics with Nb_ss_ and Nb_5_Si_3_.

Material	Parameter					
	ΔH_mix_ /(kJ/mol)	ΔS_mix_ (J/molK)	VEC	δ	Δχ	Ω ^+^
Nb-silicide based alloys [62]	−32.7 to –44.8	8.3–14.7	4.37–4.9	8.1–14.3	0.12–0.237	0.57–0.95
Nb_ss_ in Nb-silicide based alloys [38]	−2 to –15.9	5.8–14.5	4.4–5.4	2.4–9.7	0.039–0.331 with a gap in the range 0.13 to 0.179	1.55–8.9
Eutectics with Nb_ss_ and Nb_5_Si_3_	−25.5 to –41.9	4.7–15	4.33–4.89	6.23–9.44	0.118–0.248 with a gap in the range 0.164 to 0.181	0.38–1.35

+ The capital letter Q was used instead of Ω for the ratio T_m_ΔS_mix_ /|ΔH_mix_|in [38].
